# Supplementary material for: Exploring the mobilome and resistome of Enterococcus faecium in a One Health context across two continents
Source: Microb Genom. 2022 Sep 21;8(9):mgen000880. doi: 10.1099/mgen.0.000880 (PMC9676038; doi:10.1099/mgen.0.000880)
Supplement: Supplementary material 1 [file mgen-8-880-s001.pdf]

# List of Supplementary Figures

S1 Clade-specific genome characteristics. . . . . . . . . . . . . . . . . . 2
S2 Clade-specific feature distributions. . . . . . . . . . . . . . . . . . . . 3
S3 Feature clustergrams, panels A-B . . . . . . . . . . . . . . . . . . . . 4
S3 Feature clustergrams, panels C-D . . . . . . . . . . . . . . . . . . . . 5
S3 Feature clustergrams, panels E-F . . . . . . . . . . . . . . . . . . . . 6
S4 BayesTraits *p* value density plots, panels A-B . . . . . . . . . . . 7
S4 BayesTraits *p* value density plots, panels C-D . . . . . . . . . . . 8
S4 BayesTraits *p* value density plots, panels E-F . . . . . . . . . . . 9
S5 Macrolide resistance detailed plots. . . . . . . . . . . . . . . . . . . 10
S6 *tet* gene detailed plots. . . . . . . . . . . . . . . . . . . . . . . . . . 11
S7 *cop* gene detailed plots. . . . . . . . . . . . . . . . . . . . . . . . . . 12
S8 Virulence factor genes detailed plots. . . . . . . . . . . . . . . . . . 13
S9 *pil* gene detailed plots. . . . . . . . . . . . . . . . . . . . . . . . . . . 14
S10 Likelihood ratios of co-evolution between AMR genes and sampling geography. . . . . . . . . . . . . . . . . . . . . . . . . . 15

# List of Supplementary Table Captions

S1 List of genomes with metadata and quality-control metrics. . . . 16
S2 ANOVA and post-hoc testing of feature associations with sample metadata. . . . . . . . . . . . . . . . . . . . . . . . . . . . . . . . 16
S3 Genomic feature frequencies. . . . . . . . . . . . . . . . . . . . . . . 16
S4 Genomic feature distribution by sampling location and genome type. . . . . . . . . . . . . . . . . . . . . . . . . . . . . . . . . . . 16
S5 Genomic features distribution by genome type. . . . . . . . . . . 16
S6 BayesTraits likelihood ratio and p values for features against genome metadata. . . . . . . . . . . . . . . . . . . . . . . . . . . . 17
S7 Genomic feature colocalization information. . . . . . . . . . . . 17
S8 Acronyms for the data categories used in the manuscript. . . . . 17

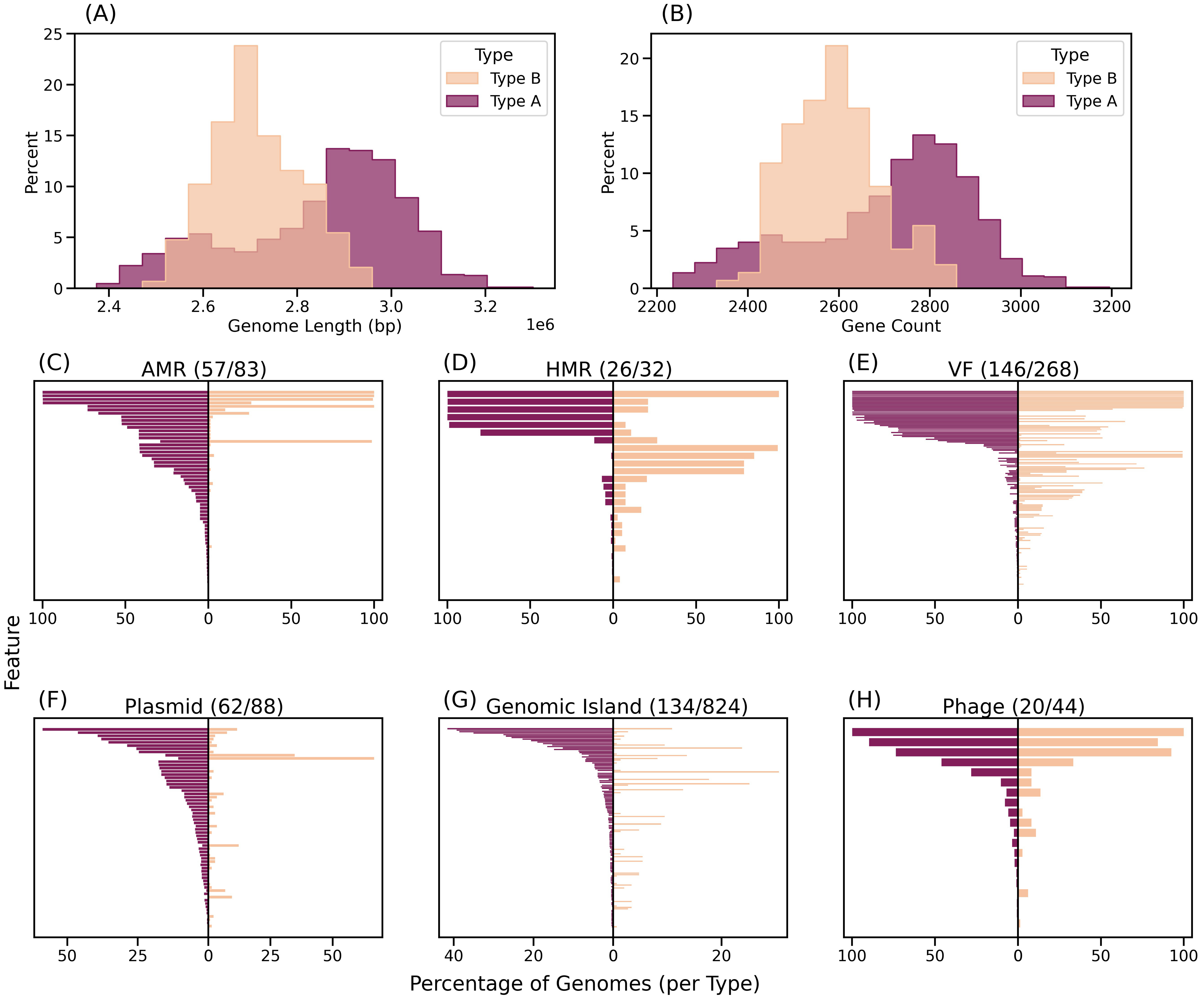

(A)
Type
Type B
Type A
Percent
Genome Length (bp)
1e6
(B)
Type
Type B
Type A
Percent
Gene Count
(C)
AMR (57/83)
(D)
HMR (26/32)
(E)
VF (146/268)
Feature
(F)
Plasmid (62/88)
(G)
Genomic Island (134/824)
(H)
Phage (20/44)
Percentage of Genomes (per Type)

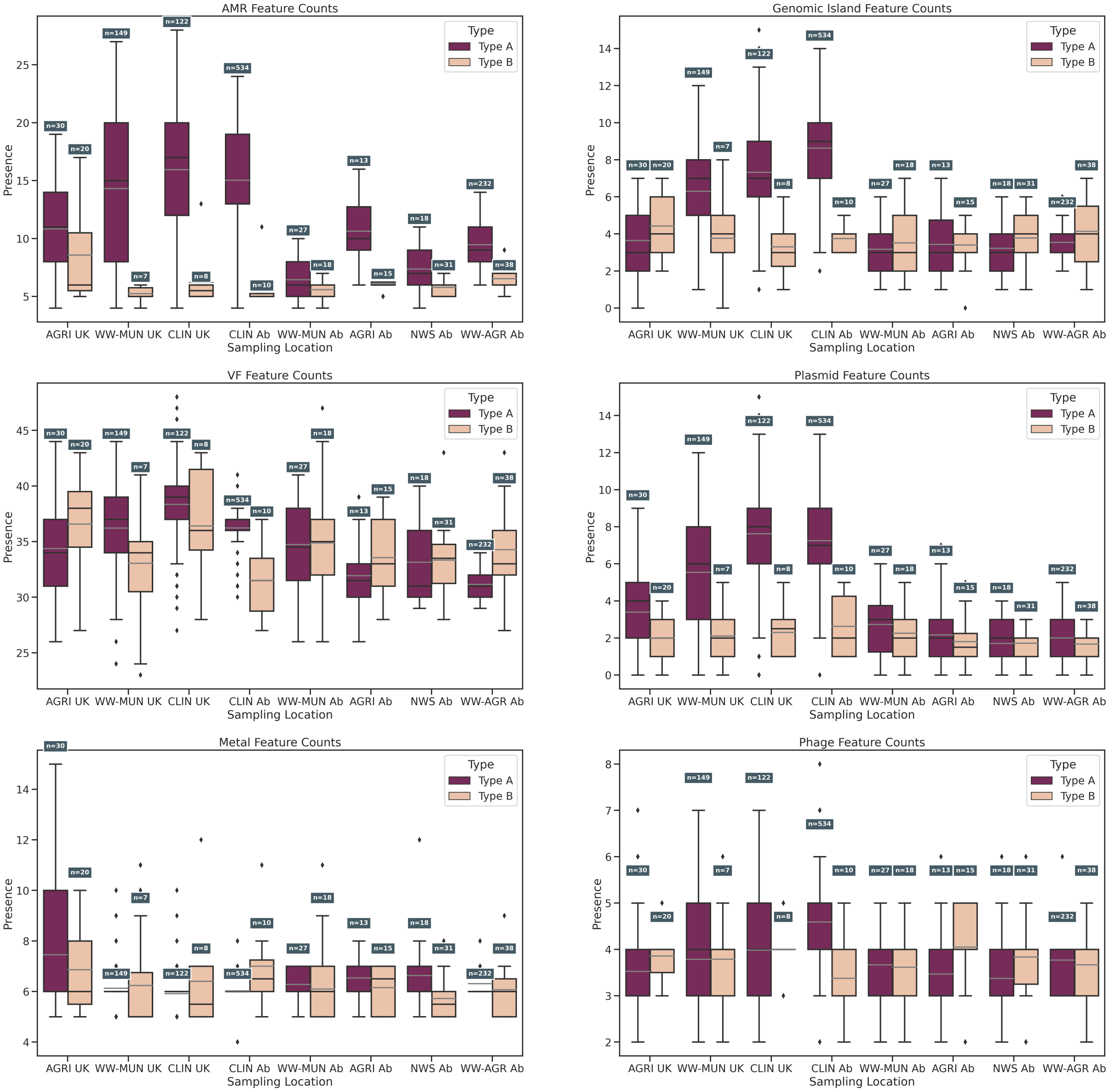


Figure S2: Clade-specific abundance of features by sampling location. X-axis groups bars by habitat-geography sampling location. Y-axis indicates the number of unique features of a given category found per genome. Bars indicate quartiles. Points/diamonds are considered to be outliers if they fall outside 1.5 × the interquartile range. Grey bars indicate mean values. Text boxes indicate the number of genomes within a given bar.

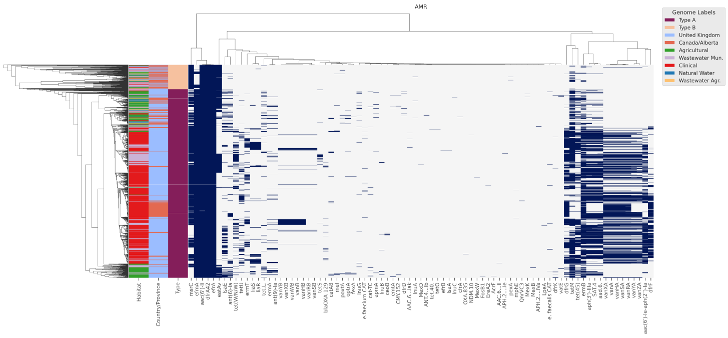


(a) AMR determinants.

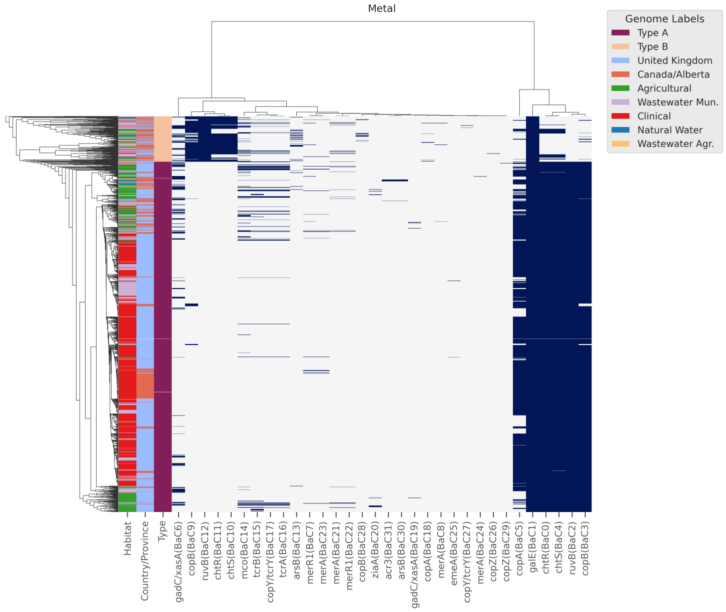


(b) HMR Determinants.

Figure S3: Feature clustergrams of target genes and MGEs. Rows indicate genomes, sorted by topology of the core genome maximum likelihood phylogenetic tree. Row colors indicate genome habitats, country of origin, and type assignment from left to right. Columns are clustered using single-linkage agglomerative clustering with Manhattan distance over the presence/absence vectors. (A) AMR determinants. (B) HMR determinants. (C) Virulence factors. (D) Plasmid clusters. (E) Genomic islands. (F) Phages.

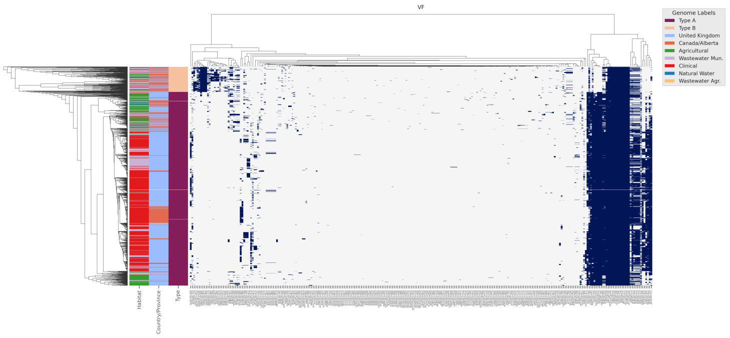


(c) Virulence factors.

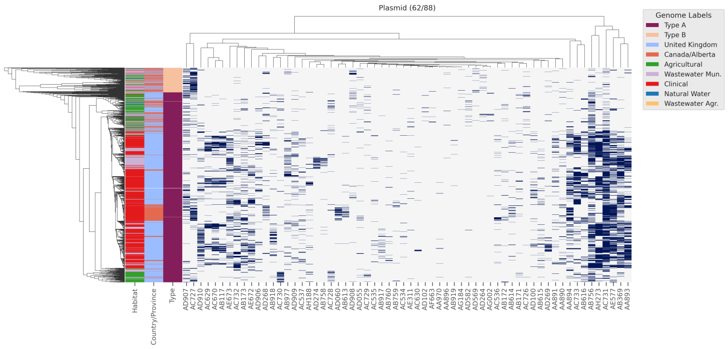


(d) Plasmid clusters.

Figure S3: Feature clustergrams of target genes and MGEs. Rows indicate genomes, sorted by topology of the core genome maximum likelihood phylogenetic tree. Row colors indicate genome habitats, country of origin, and type assignment from left to right. Columns are clustered using single-linkage agglomerative clustering with Manhattan distance over the presence/absence vectors. (A) AMR determinants. (B) HMR determinants. (C) Virulence factors. (D) Plasmid clusters. (E) Genomic islands. (F) Phages.

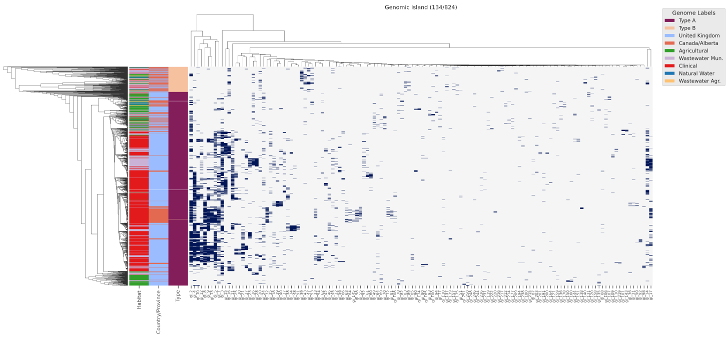


(e) Genomic Islands.

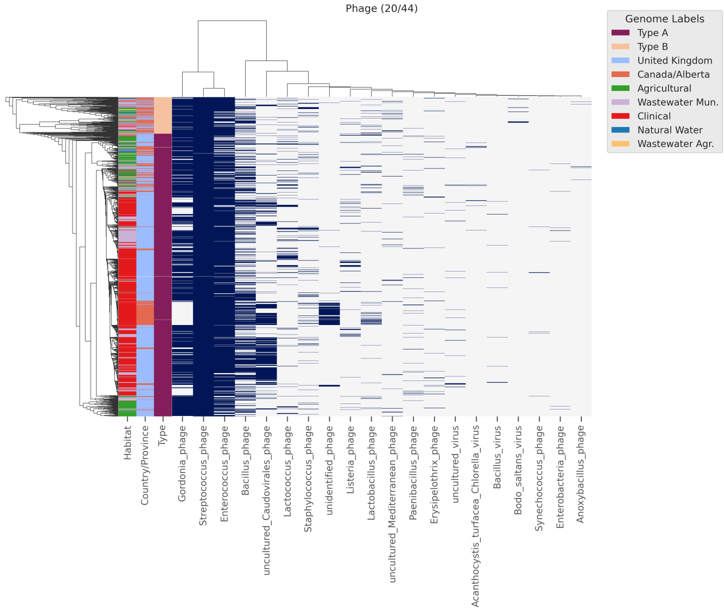


(f) Phages.

Figure S3: Feature clustergrams of target genes and MGEs. Rows indicate genomes, sorted by topology of the core genome maximum likelihood phylogenetic tree. Row colors indicate genome habitats, country of origin, and type assignment from left to right. Columns are clustered using single-linkage agglomerative clustering with Manhattan distance over the presence/absence vectors. (A) AMR determinants. (B) HMR determinants. (C) Virulence factors. (D) Plasmid clusters. (E) Genomic islands. (F) Phages.

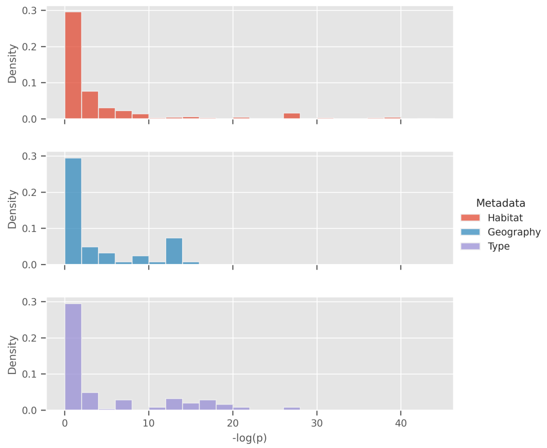


(a) AMR determinant $p$ value distributions.

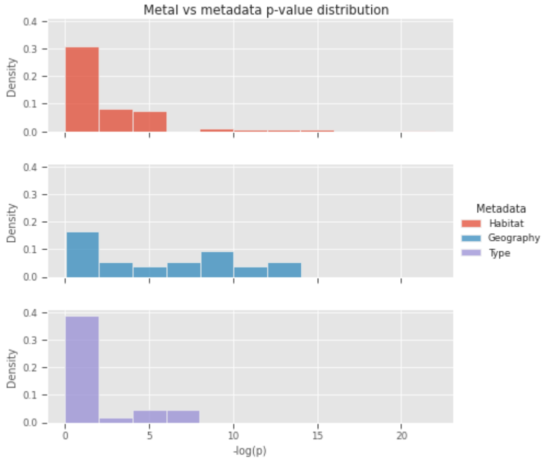


(b) HMR determinant $p$ value distributions.

Figure S4: Density histograms of BayesTraits $p$-values testing the hypothesis that feature evolution is dependant on habitat category, geographical sampling location, and assigned type. Y axis indicates the total are under the curve for each histogram. X axis indicates $-log(p)$. (A) AMR determinant genes. (B) HMR determinant genes. (C) Virulence factors. (D) Plasmid cluters. (E) Genomic islands. (F) Phages.

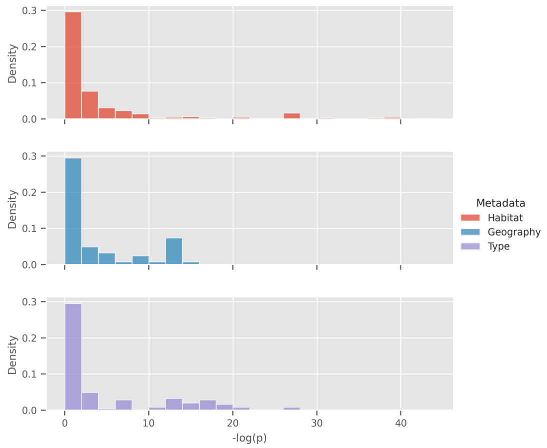


(c) Virulence factor $p$ value distributions.

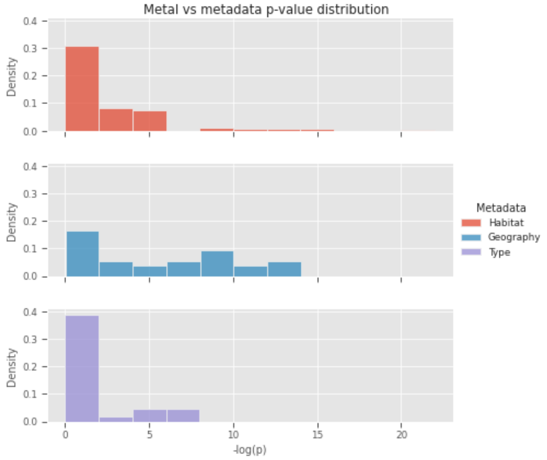


(d) Plasmid cluster $p$ value distributions.

Figure S4: Density histograms of BayesTraits $p$-values testing the hypothesis that feature evolution is dependant on habitat category, geographical sampling location, and assigned type. Y axis indicates the total are under the curve for each histogram. X axis indicates $-log(p)$. (A) AMR determinant genes. (B) HMR determinant genes. (C) Virulence factors. (D) Plasmid cluters. (E) Genomic islands. (F) Phages.

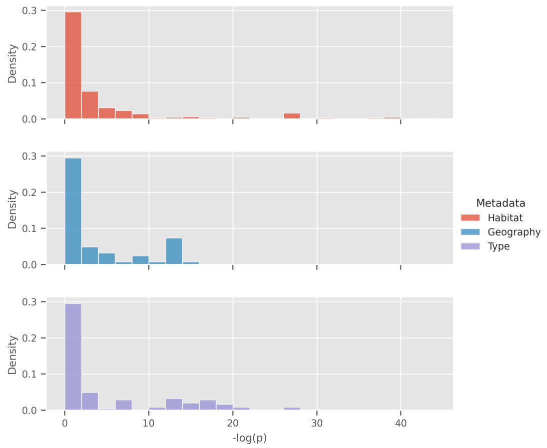


(e) Genomic island $p$ value distributions.

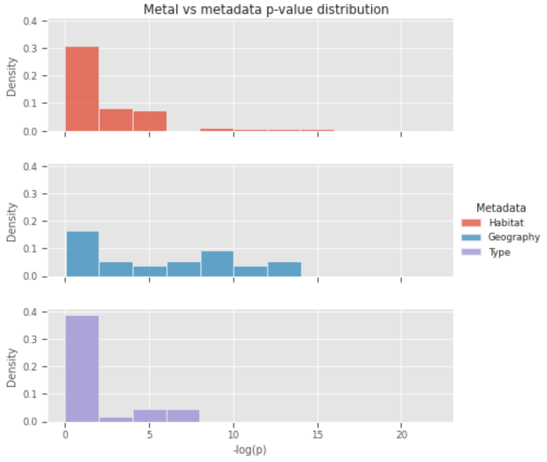


(f) Phage $p$ value distributions.

Figure S4: Density histograms of BayesTraits $p$-values testing the hypothesis that feature evolution is dependant on habitat category, geographical sampling location, and assigned type. Y axis indicates the total are under the curve for each histogram. X axis indicates $-log(p)$. (A) AMR determinant genes. (B) HMR determinant genes. (C) Virulence factors. (D) Plasmid cluters. (E) Genomic islands. (F) Phages.

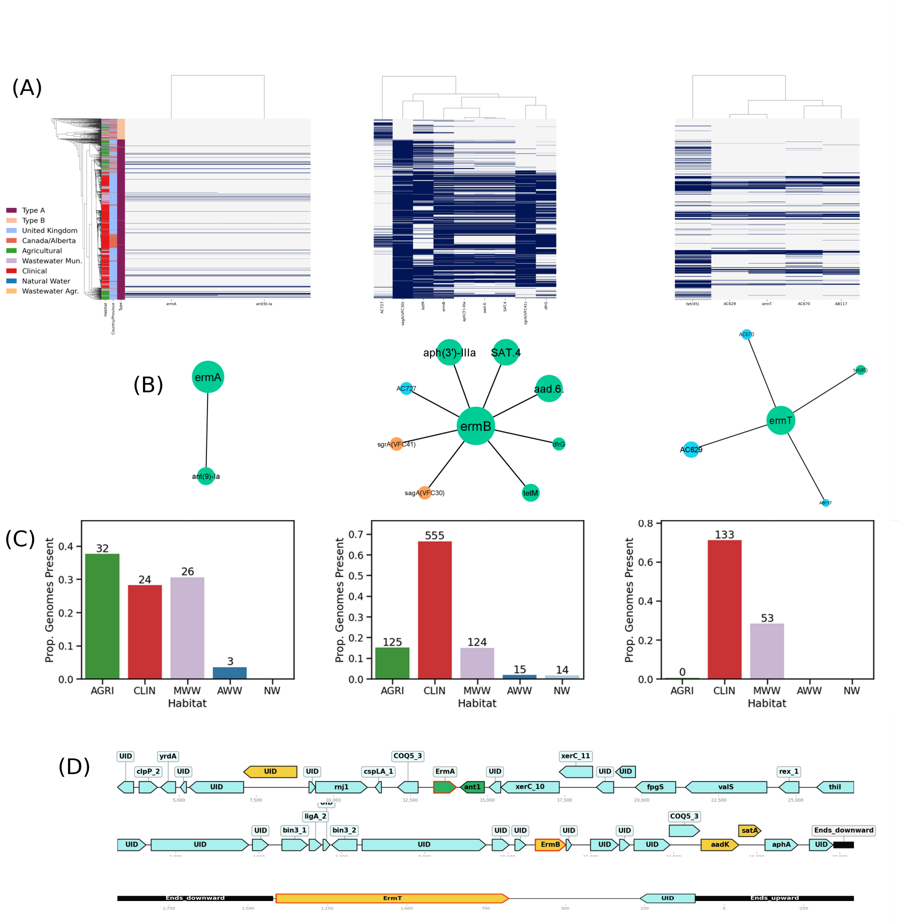


Figure S5: Statistical associations and physical localization of macrolide-resistance genes *ermA*, *ermB*, and *ermT*. (a) phylogenetic distribution of erm genes and associated genes with associated likelihood ratio $\geq 100$. (b) Statistical association networks of *erm* genes. (c) Distribution of genes by habitat. (d) Examples of gene order on identified contigs. Gene and MGE colours are consistent with those in Figure 2. Green genes correspond to perfect matches with reference genes in the CARD database, yellow genes are Strict hits.

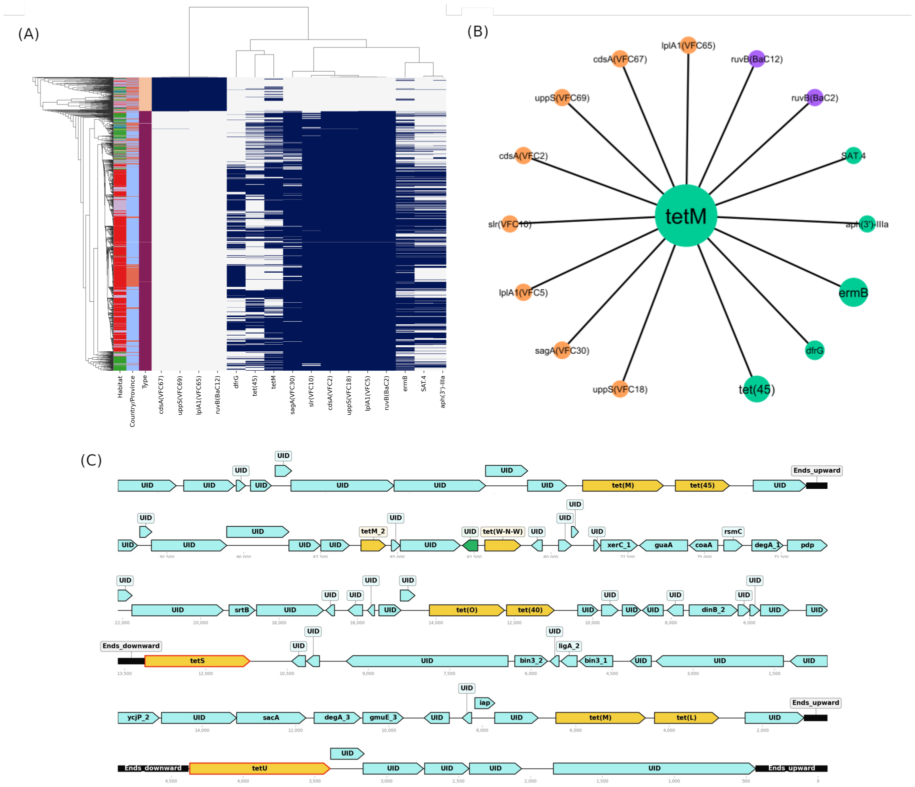


Figure S6: Associations of tetM with other features and example neighbourhoods of tet genes. (a) phylogenetic distribution of tetM and genes with associated likelihood ratio $\geq 100$. (b) Statistical association networks of tetM. (c) Co-localization of tetM with other tetracycline-resistance genes, and example neighbourhoods of other tet genes. Gene and MGE colours are consistent with those in Figure 2. Green genes correspond to perfect matches with reference genes in the CARD database, yellow genes are Strict hits.

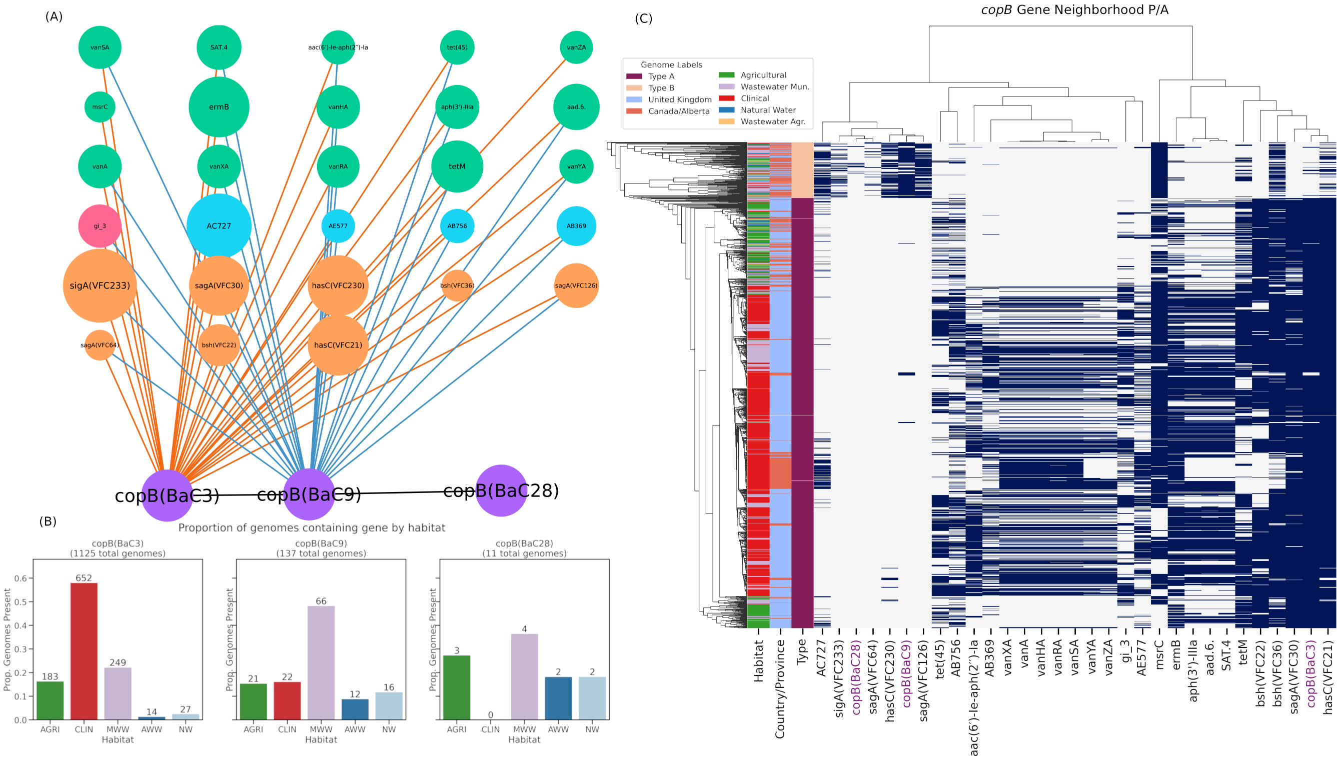


Figure S7: Distribution and associations of *copB* genes. (A) Statistical association network of *copB* genes with other features. Gene and MGE colours are consistent with those in Figure 2. (B) Habitat distributions of *copB* genes. (C) Phylogenetic distribution of genes and other features with an associated likelihood ratio $\geq 50$.

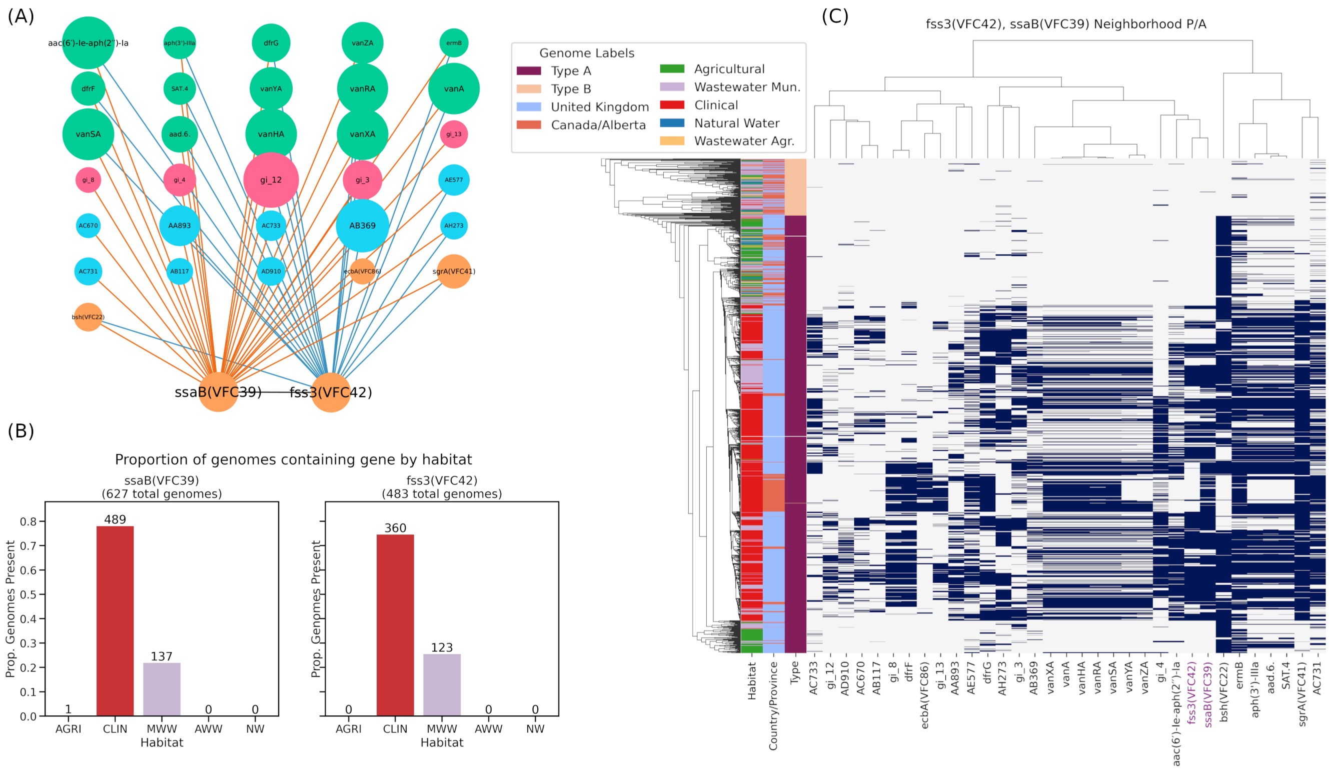


Figure S8: Distribution and associations of *ssaB* and *fss3* genes. (A) Statistical association network of *ssaB* and *fss3* genes with other features. Gene and MGE colours are consistent with those in Figure 2. (B) Habitat distributions of *ssaB* and *fss3* genes. (C) Phylogenetic distribution of genes and other features with an associated likelihood ratio $\geq 100$.

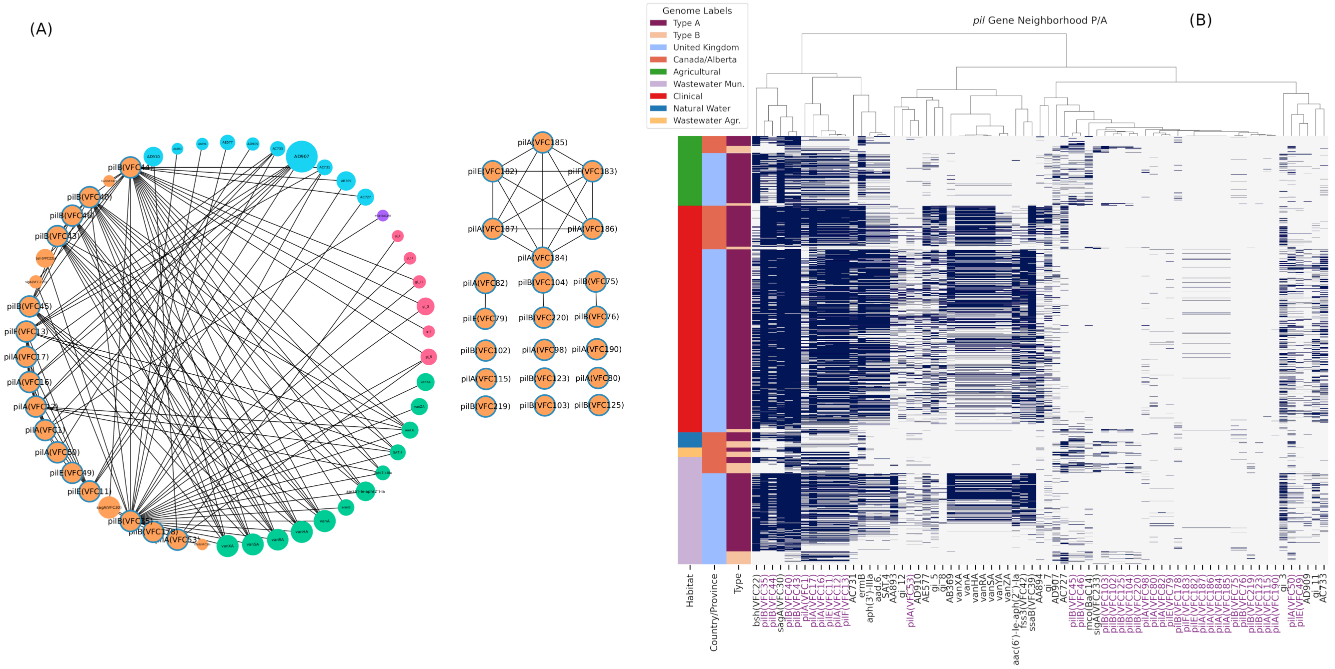

Figure S9: Distribution and associations of *pil* genes. (A) Statistical association network of *pil* genes with other features. *pil* genes are circled in blue. Gene and MGE fill colours are consistent with those in Figure 2. (B) Presence/absence of *pil* genes. Rows indicate genomes and are sorted by habitat. Genes include *pil* genes and genes/MGEs that associate with any *pil* gene with a likelihood ratio $\geq 75$.

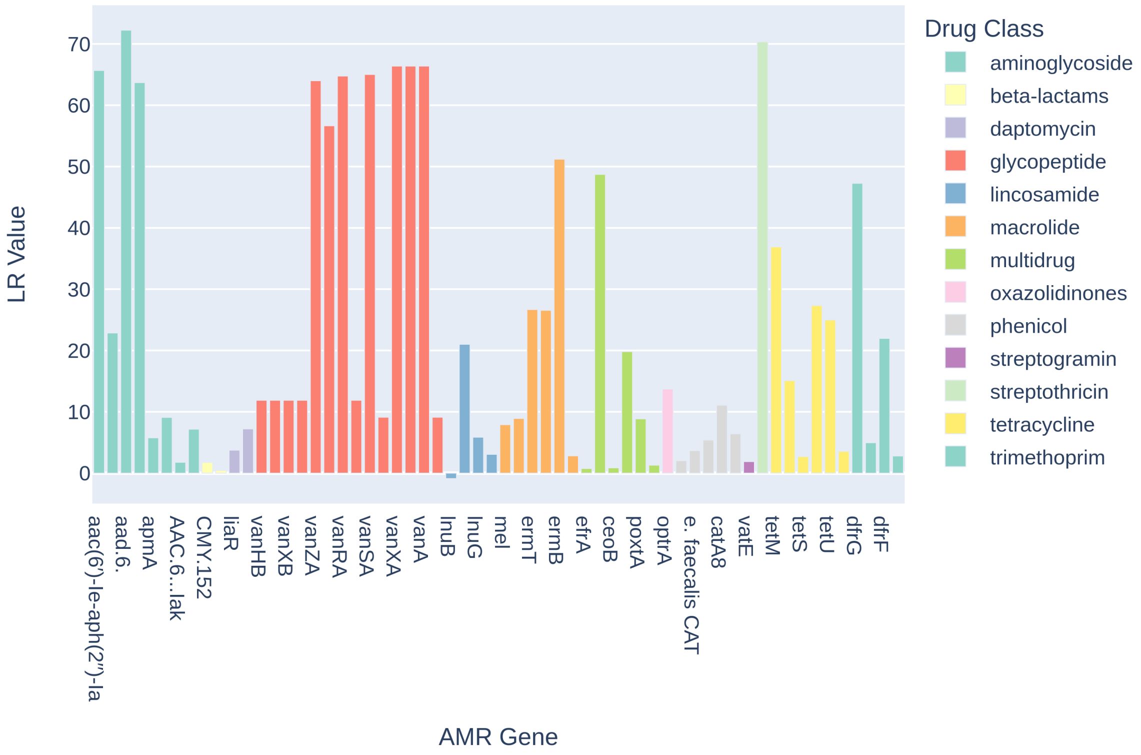


Figure S10: Likelihood ratios of co-evolution between AMR genes and sampling geography. Ratios indicate the likelihood that AMR feature distribution fits a co-evolution model with geography label more strongly than an independent evolution model. Bars are colored by drug class of resistance conferred.

## 2 Supplementary Table Captions

Table S1: List of genomes with metadata and quality-control metrics.

Table S2: ANOVA and post-hoc testing of feature associations with sample metadata. Sheet (a) contains ANOVA results as reported in tables 1 and 2, color-coded by $p$-value. Sheets (b) - (g) contain Tukey's HSD post-hoc statistics for each ANOVA group for AMR (b), HMR (c), VF (d), Plasmid (e), GI (g), and Phage (g) features.

Table S3: Genomic feature frequencies. Each sheet describes all features detected of a specific category: AMR (a), HMR (b), VF (c), Plasmid (d), GI (e), and Phage (f). The "count" column indicates the number of genomes in which a feature was detected. The "percent" column indicated the proportion of genomes in which a feature was detected. The "gene_type" column indicates whether a feature is designated as a core, shell, or cloud.

Table S4: Genomic feature distribution by sampling location and genome type. Tables Indicate the total number of unique features in each category (AMR, HMR, VF, Plasmid, GI, Phage) detected within genomes divided by their sampling location and type assignment. Reports the mean and standard deviation of the number of unique features per category.

Table S5: Genomic features distribution by genome type. Sheets a-f contain information for AMR (a), HMR (b), VF (c), Plasmid (d), GI (e), and Phage (f) features, respectively. Each sheet summarizes the presence of individual genomic features by genome type. Type A percent indicates the percentage of type A genomes which contain a feature. Type B percent indicates the percentage of type B genomes which contain a feature. Percentage Difference indicates the delta between the two percentages. Different shades of color intensity represent the percentage of genomes per type carrying the feature (red) or the percentage difference (green).

Table S6: BayesTraits likelihood ratio and $p$ values for features against genome metadata. The LR value indicates the ratio of likelihood of a dependent vs an independent evolutionary model, with higher values indicating higher likelihood of dependent evolution. $p$ values are calculated by treating the LR value as an approximate Chi-squared statistic. "n Genomes Present" indicates the number of genomes within a given metadata category that contain the feature. "Proportion Present" indicates the proportion of genomes within a given metadata category that contain the feature. Sheets a-f contain data for AMR (a), HMR (b), VF (c), Plasmid (d), GI (e), and Phage (f) features, respectively.

Table S7: Genomic feature colocalization information. Sheet (a) contains localization information for AMR, HMR, and VF genes. "Start" indicates the position of the feature in nucleotide coordinates with respect to the genome assembly. "Plasmid_cluster" indicates the plasmid cluster ID if a feature falls on a contig which was classified as a plasmid cluster. "GI_cluster" indicates the genomic island ID if a feature falls within the same coordinates as a predicted genomic island. "Phage_cluster" indicates the phage cluster ID if a feature falls within the same coordinates as a predicted phage. "Chromosome" indicates whether a feature was found on a chromosomal contig, with 1 indicating "yes" and 0 indicating "no". Sheets b-d contain pivot tables indicating the genomic features identified within each mobile genetic element. Each sheet summarizes the number of AMR, HMR, and VF features found within a given MGE as well as a description of the specific features found. Sheet (b) contains information for plasmid clusters, (c) for genomic islands, and (d) for phage clusters. Sheet (e) summarizes the frequency and proportion of features within chromosomal contigs and/or MGEs.

Table S8: Acronyms for the data categories used in the manuscript.
